# Drug-Induced Hypersensitivity Syndrome Due to Long-Term Usage of Methotrexate: A Case Report

**DOI:** 10.7759/cureus.58659

**Published:** 2024-04-20

**Authors:** Taichi Fujimori, Chiaki Sano, Ryuichi Ohta

**Affiliations:** 1 Community Care, Unnan City Hospital, Unnan, JPN; 2 Community Medicine Management, Shimane University Faculty of Medicine, Izumo, JPN

**Keywords:** drug-induced hypersensitivity syndrome, pneumonitis, prednisolone, family medicine, rural, rheumatoid arthritis, methotrexate

## Abstract

Methotrexate (MTX), a cornerstone treatment for rheumatoid arthritis (RA), is associated with drug-induced hypersensitivity syndrome (DIHS), including rare instances of methotrexate-induced pneumonitis. We report a significant case of a 65-year-old woman with RA, treated with MTX for over two decades, who presented with fever, headache, nausea, and malaise and was later diagnosed with DIHS, manifesting as pneumonitis and hepatosplenomegaly. Despite initial suspicion of bacterial pneumonia, her condition deteriorated, leading to the consideration of DIHS. The diagnosis was confirmed through a drug lymphocyte stimulation test (DLST), and she responded well to prednisolone. This case underlines the complexity of long-term MTX therapy, emphasizing the need for vigilance towards DIHS even after years of treatment. The findings prompt a reconsideration of ongoing treatments for RA, particularly in settings where long-term MTX use is prevalent. Early intervention and diagnostic tests like the DLST are crucial for preventing severe outcomes. This case adds to the growing evidence of MTX's potential for causing DIHS even in long-term usage. It stresses the importance of balancing therapeutic benefits with the risks of significant adverse reactions in stable RA patients.

## Introduction

Methotrexate (MTX) is known as a drug that causes drug-induced hypersensitivity syndrome (DIHS). One expression of DIHS is drug-induced pneumonitis [1]. During the diagnosis of drug-induced pneumonitis, it is considered if pneumonia occurs during the administration of the causative drug [1]. Symptoms include coughing, difficulty breathing, fever, and abnormal breath sounds. Laboratory tests show hypoxemia and elevated inflammatory markers, and elevated KL-6 and SA/B levels are said to be helpful in diagnosis [2]. A drug-induced lymphocyte stimulation test (DLST) may be used to confirm the causative drug [3]. Methotrexate has various effects on the inflammatory mechanisms of the human body, such as suppressing the secretion of tumor necrosis factor-alpha (TNF-α) and causing pneumonitis.

A pharmacological mechanism is involved in MTX-induced DIHS. Methotrexate reduces the activity of rheumatoid arthritis (RA) by suppressing antibody production, lymphoid proliferation, angiogenesis, synovial hyperplasia, neutrophil migration to inflammatory sites, interleukin-1 production, and collagenase production [3]. Pneumonia caused by MTX is called methotrexate-induced pneumonitis [3]. Although this mechanism has yet to be completely elucidated, several hypotheses have been proposed. Methotrexate alters the immune system, including interleukin-8, which can induce autoimmune and hypersensitivity reactions and lead to pneumonia [4]. Methotrexate can directly damage lung tissue and cause inflammatory responses and tissue damage [5]. Methotrexate causes oxidative stress, promoting inflammation and damage to lung tissue [3]. Drug-induced hypersensitivity syndrome with MTX can also result in pneumonia.

Although most MTX-related DIHS is said to occur within a few months, it is uncommon to develop it after long-term administration, such as years and decades [5,6]. Here, we experienced a case of MTX-related DIHS that occurred after more than 20 years of MTX administration. During the detailed examination of the fever of unknown origin, we detected hepatosplenomegaly and diffusely distributed infiltrative shadows in the lungs, so we considered the possibility of DIHS due to MTX, and a thorough examination led to a diagnosis. Through the experience of this case, we discuss the possibility that DIHS may occur due to the long-term use of MTX, as well as a smooth diagnostic method for it.

## Case presentation

A 65-year-old woman presented to a remote community hospital with complaints of fever, headache, nausea, and malaise. She had been taking MTX (12 mg) weekly for several years for RA. She had experienced fever and general malaise starting two weeks before her visit, with a tendency to develop fever at night. On the day before her visit, she had a fever of 38.5°C and took 500 mg of acetaminophen for relief. She visited the hospital due to the onset of a headache and nausea on the day of her visit. Her medical history included rheumatoid arthritis, functional gastrointestinal disorders, and osteoporosis. She had normal lung function test results one year before the admission. Her medication history included MTX of 12 mg weekly, iguratimod of 25 mg daily, folic acid of 5mg weekly, and vonoprazan of 10 mg daily.

Upon arrival, her consciousness was clear, and her vital signs were as follows: body temperature of 38.1℃, blood pressure of 96/61 mmHg, pulse rate of 97/min, respiratory rate of 21/min, and oxygen saturation (SpO2) of 97% in room air. Physical examination showed mild conjunctival congestion and crackles at the base of both lungs, with no other significant findings in the head, neck, chest, abdomen, or skin. Blood tests revealed elevated white blood cells and C-reactive protein, indicating an inflammatory state and increased lactate dehydrogenase (LDH) (Table 1).

**Table 1:** Initial laboratory data of the patient Ig: immunoglobulin

Parameter	Level	Reference range
White blood cells	13.00	3.5–9.8 × 10^3^/μL
Neutrophils	72.5	%
Lymphocytes	12.6	%
Hemoglobin	12.2	11.3–15.2 g/dL
Hematocrit	36.9	34–43%
Mean corpuscular volume	96.8	82–101 fl
Platelets	21.1	13.0–36.9 × 10^4^/μL
Erythrocyte sedimentation rate	71	3–15 mm/hour
Total protein	6.9	6.6–8.1 g/dL
Albumin	3.4	3.9–4.9 g/dL
Total bilirubin	0.6	0.2–1.2 mg/dL
Aspartate aminotransferase	36	8–38 IU/L
Alanine aminotransferase	21	4–44 IU/L
Lactate dehydrogenase	299	106–211 U/L
Blood urea nitrogen	8.2	8–20 mg/dL
Creatinine	0.58	0.40–1.10 mg/dL
Serum sodium (Na)	138	135–147 mEq/L
Serum potassium (K)	3.8	3.3–4.8 mEq/L
Serum chloride (Cl)	103	98–108 mEq/L
C-reactive protein (CRP)	8.27	<0.30 mg/dL
IgG	1499	870–1700 mg/dL
IgM	51	35–220 mg/dL
IgA	182	110–410 mg/dL
Urine test	-	-
Leukocyte	Negative	Negative
Protein	Negative	Negative
Blood	Negative	Negative

**Figure 1:**
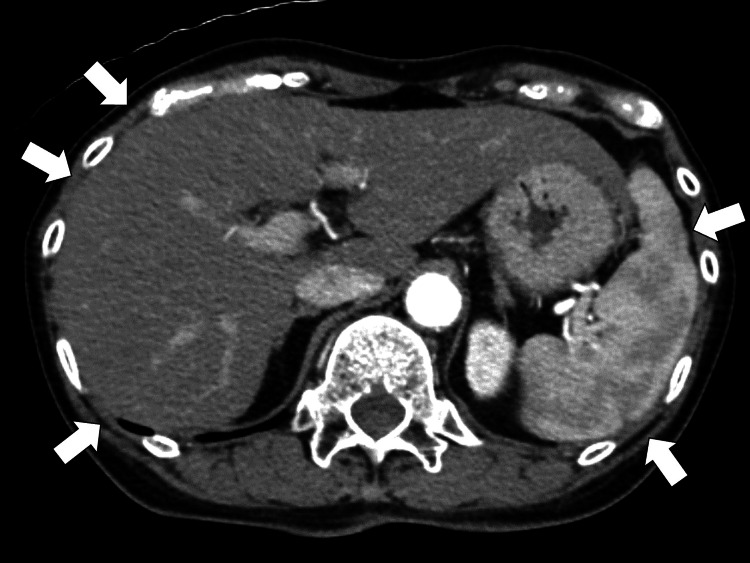
Contrast-enhanced computed tomography scans from the chest to the pelvis show hepatosplenomegaly without lymphadenopathy (white arrows).

There was no significant increase in eosinophils or immunoglobulin E. Contrast-enhanced computed tomography (CT) scans from the chest to the pelvis showed infiltrative shadows in both lungs and hepatosplenomegaly but no apparent lymph node swelling (Figure 1).

**Figure 2:**
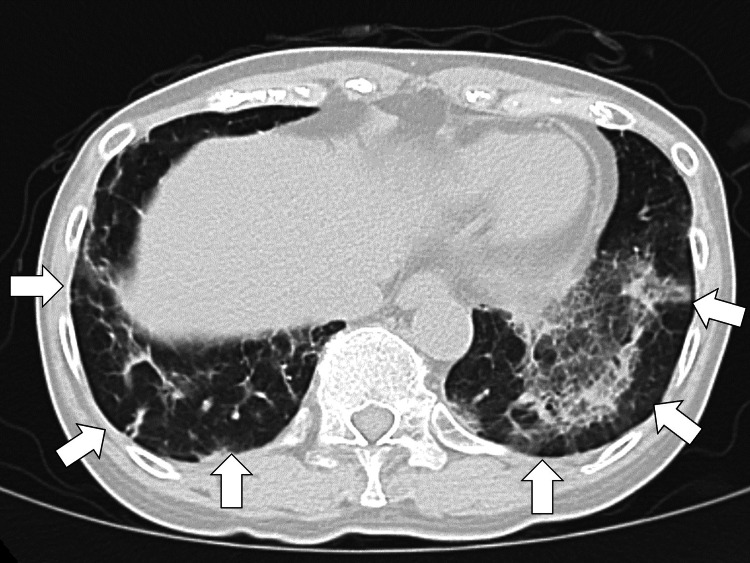
A follow-up chest computed tomography shows an exacerbation of the infiltrative shadows in both lungs (white arrows).

Considering the immunosuppressive state due to RA treatment, bacterial pneumonia was suspected from the first day of hospitalization, and ceftriaxone 2 g/day was initiated. However, the fever did not subside, and the general malaise worsened. On the sixth day of hospitalization, she developed respiratory distress, and a follow-up chest CT showed an increase in the infiltrative shadows in both lungs (Figure 2).

An increase in KL-6 (742 U/mL, reference range: 105.3 to 401.2) was also observed. Blood cultures were negative, suggesting a low likelihood of bacterial infection. Given the course of her symptoms, the possibility of autoimmune disease or systemic inflammation due to medication was considered. Long-term use of MTX raised the possibility of DIHS, so additional testing with a DLST was conducted, considering conjunctival congestion and hepatosplenomegaly. Given the progressive nature of her condition and the potential for severity, treatment with prednisolone at 1 mg/kg was initiated.

Subsequently, her fever quickly lowered, and her malaise gradually improved. On the tenth day of hospitalization, the DLST results showed high lymphocyte reactivity to MTX (1,189 cpm, reference range: 156<). She was discharged home on the 21st day of hospitalization without symptoms. Seven days after discharge, a follow-up CT scan for pulmonary lesions showed that the infiltrative shadows in the lung had disappeared. Prednisolone was tapered by 10 mg/week and successfully discontinued after two months.

## Discussion

In this case report, we encounter a case where an elderly patient with RA developed DIHS to MTX after prolonged use, leading to progressive lung injury and hepatosplenomegaly. This case sheds light on the complexities associated with long-term MTX therapy, traditionally employed for its efficacy in controlling RA but also known for potential adverse reactions. The nature of DIHS observed in this patient underscores the critical need for awareness regarding the possible spectrum of MTX-induced side effects, which may include both acute and delayed hypersensitivity reactions of DIHS.

This case report has documented MTX's capability to provoke DIHS not only in the short term but also with prolonged exposure. Studies have shown that while MTX is a cornerstone in RA treatment due to its anti-inflammatory and immunomodulatory effects, its association with adverse reactions such as pulmonary toxicity, hepatotoxicity, and systemic inflammation is increasingly recognized [7, 8]. These adverse effects underscore the drug's potential to elicit a wide range of immune responses, leading to conditions as severe as the one described in our patient.

Recognizing signs of hepatosplenomegaly and multiple organ inflammatory reactions from DIHS is paramount for effectively diagnosing such conditions. These findings are significant because they hint at underlying systemic reactions in DIHS that could be triggered by medications like MTX. The literature suggests that the presence of these symptoms can often predate the diagnosis of DIHS, thereby serving as critical indicators for clinicians to reconsider ongoing treatments [9]. Cases have been reported where onset occurred more than eight years after administration [10, 11]. Risk factors identified include being aged 60 years or older, diabetes mellitus, hypoalbuminemia, a history of disease-modifying antirheumatic drug (DMARD) use, renal dysfunction, male gender, the severity of autoimmune diseases, and a history of pre-existing lung disease [12-15]. The principle of treatment for DIHS, including drug-induced pneumonia, is discontinuation of the causative drug, which alone can lead to improvement. If inflammation is severe, prednisolone should also be used [12-15].

Moreover, the importance of timely intervention, especially in a rural setting, needs to be addressed. Rural general physicians, who might be the first point of contact for patients experiencing DIHS to MTX, must be vigilant about the possibility of such occurrences [16, 17]. The recommendation to promptly check the DLST for MTX is supported by evidence suggesting its utility in identifying DIHS indicative of a hypersensitivity reaction [9]. Furthermore, the early withdrawal of MTX, accompanied by the initiation of corticosteroid therapy, is crucial for preventing the progression of DIHS, as evidenced by the positive outcome in this case [9]. This approach aligns with current guidelines that advocate for the swift management of DIHS to mitigate the risk of long-term complications.

## Conclusions

This case illustrates the complexity of managing DIHS, particularly in patients with RA undergoing long-term MTX therapy. It highlights the need for continuous monitoring for DIHS, the importance of early diagnostic measures like DLST, and the critical role of timely intervention to prevent severe outcomes. The insights from this case should inform clinical practice and future research, emphasizing the necessity of balancing therapeutic benefits with the potential for significant DIHS in managing chronic conditions such as RA and the consideration of reducing the dose of MTX in stable RA patients.
